# High virulence is associated with pathogen spreadability in a songbird–bacterial system

**DOI:** 10.1098/rsos.220975

**Published:** 2023-01-11

**Authors:** Dana M. Hawley, Courtney A. Thomason, Matt A. Aberle, Richard Brown, James S. Adelman

**Affiliations:** ^1^ Department of Biological Sciences, Virginia Tech, Blacksburg, VA 24061-0131, USA; ^2^ Department of Biological Sciences, The University of Memphis, Memphis, TN 38152, USA

**Keywords:** house finch, inflammation, *Mycoplasma gallisepticum*, transmission, virulence evolution

## Abstract

How directly transmitted pathogens benefit from harming hosts is key to understanding virulence evolution. It is recognized that pathogens benefit from high within-host loads, often associated with virulence. However, high virulence may also directly augment spread of a given amount of pathogen, here termed ‘spreadability’. We used house finches and the conjunctival pathogen *Mycoplasma gallisepticum* to test whether two components of virulence—the severity of conjunctival inflammation and behavioural morbidity produced—predict pathogen spreadability. We applied ultraviolet powder around the conjunctiva of finches that were inoculated with pathogen treatments of distinct virulence and measured within-flock powder spread, our proxy for ‘spreadability’. When compared to uninfected controls, birds infected with a high-virulence, but not low-virulence, pathogen strain, spread significantly more powder to flockmates. Relative to controls, high-virulence treatment birds both had more severe conjunctival inflammation—which potentially facilitated powder shedding—and longer bouts on feeders, which serve as fomites. However, food peck rates and displacements with flockmates were lowest in high-virulence treatment birds relative to controls, suggesting inflammatory rather than behavioural mechanisms likely drive augmented spreadability at high virulence. Our results suggest that inflammation associated with virulence can facilitate pathogen spread to conspecifics, potentially favouring virulence evolution in this system and others.

## 1. Introduction

Pathogens that rely on host mobility to spread, yet are also sufficiently virulent to cause lethargy or death in their hosts, present an apparent evolutionary paradox [1,2]. Theoretical models of virulence (defined here as the pathogen contribution to infection-induced morbidity and mortality) helped resolve this paradox by demonstrating that, despite its presumed fitness costs, high virulence can still be favoured for directly transmitted pathogens (e.g. [1]). Most simply, high virulence can benefit pathogen fitness if the damage to hosts (e.g. inflammation, behavioural morbidity) associated with virulent infections directly augments an infected host's likelihood of transmission. For example, the symptoms associated with the severe tissue inflammation (e.g. coughing, diarrhea, weeping lesions) caused by some virulent pathogens can facilitate the exit of live pathogen from hosts, the deposition of pathogen onto surfaces that serve as fomites, or even the degree of pathogen viability in the external environment [3–6], all of which could augment spread to conspecifics. Further, behaviours such as immobility that result from some virulent infections have clear potential fitness benefits for pathogens that rely on successful vector biting of hosts for spread [1]. Overall, while these direct benefits of high virulence for pathogens were first hypothesized >60 years ago [7], it is challenging to isolate such benefits from the other potential fitness benefits of high virulence that may occur simultaneously for many pathogens.

It is particularly difficult to disentangle potential direct benefits of virulence for pathogens discussed above, whereby host tissue damage or morbidity itself facilitates spread, from what we term the ‘load-dependent’ benefits of virulence that are associated with high within-host pathogen replication rates in many systems [8]. For example, under a common formulation of the seminal virulence 'trade-off' hypothesis, virulence and its associated fitness costs for pathogens arise as an unavoidable consequence of the high within-host exploitation needed for successful pathogen replication and transmission [1,9]. Under this framework, higher virulence can be favoured whenever the benefits to pathogens from high within-host exploitation (for example, by augmenting the amount of within-host pathogen available to transmit) outweigh the associated fitness costs of virulence for pathogens (i.e. fewer opportunities for transmission due to morbidity [10] and mortality [11], or other trade-offs [12]). Thus, a common but not universal assumption of this framework is that transmission benefits to virulence for pathogens are largely load-dependent (e.g. [8]). Indeed, a meta-analysis of the trade-off hypothesis found support for two core assumptions of this virulence model: among pathogen strains, positive relationships were detected between pathogen within-host replication rates and virulence, and between pathogen within-host replication and transmission rate [8]. By contrast, the potential for virulence *per se* (tissue damage, behavioural morbidity) to directly augment the ability of a pathogen strain to spread a given amount of its available within-host load to conspecifics [1], leading to potential transmission benefits above and beyond those associated with higher pathogen loads, has received relatively less attention (but see [13,14]).

**Figure 1.**
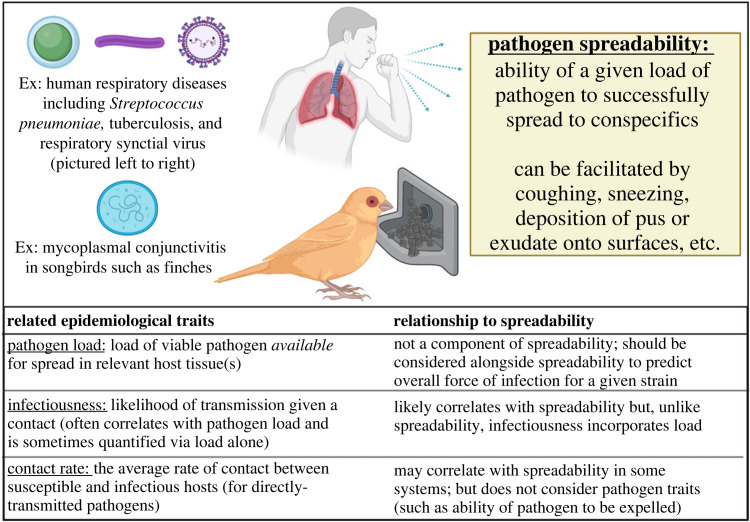
Illustration and definition of the concept of pathogen spreadability, a trait relevant for a range of directly transmitted pathogens. For context, we also define three epidemiological traits that are most related yet still distinct from spreadability, and note their expected relationship with spreadability. For most systems, pathogen spreadability would need to be combined with related traits such as pathogen load to generate robust overall estimates for a given strain's ‘force of infection’, defined generally as a product of the contact rate between susceptible and infectious hosts, and the probability that a given contact results in successful transmission [15]. While spreadability influences both components of the force of infection, the average pathogen load available for spread will also contribute to the latter component. Figure made in Biorender.

Here we aimed to explicitly test these potential direct benefits of high virulence (tissue damage, behavioural morbidity) for pathogens by quantifying the extent to which a host spreads equivalent starting amounts of an inert powder to conspecifics following inoculation with treatments of distinct virulence. By doing so, we aimed to empirically isolate one key direct benefit to high virulence for pathogens (e.g. [1,9]): the ability to spread a given amount of pathogen load to conspecifics, which we term ‘spreadability’. We note that because spreadability is defined as the ability of a strain to transmit a given load of pathogen (figure 1), in most host-pathogen systems it should be considered alongside other pathogen traits, such as a given strain's average pathogen load, to predict overall transmission potential or force of infection [15,16]. Although our metric of ‘spreadability’ shares similarities with metrics such as contact rate and ‘infectiousness’ [17], the lack of clear parallels with existing terms in the infectious disease literature led us to use a unique term (figure 1). Few empirical studies have explored the role of direct benefits of strain virulence (tissue damage, behavioural morbidity) for pathogens, but here we leveraged a system for which pathogen virulence has steadily increased in the wild [18,19], and for which prior work suggests direct benefits of virulence (in the form of tissue damage) for transmission: house finches (*Haemorhous mexicanus*) and their bacterial pathogen *Mycoplasma gallisepticum* (MG).

The severe conjunctivitis caused by MG infection in house finches significantly reduces host survival rates and resulted in notable population declines following initial pathogen emergence [20,21], suggesting that high virulence carries mortality costs for MG in this system. MG in finches is transmitted directly or via indirect contacts on environmental fomites such as bird feeders [22]; however, the fairly limited environmental viability of MG outside of the host [23] makes this pathogen highly dependent on host mobility for transmission, akin to an exclusively directly transmitted pathogen [24]. Nonetheless, following its emergence in house finches in the mid-1990s [25], MG has steadily increased in virulence on each coast of the United States [18,19]. Prior studies found that more virulent MG strains are associated with higher within-host pathogen loads [19] and higher transmission rates [24], suggesting the potential for associated benefits of virulence in terms of higher within-host pathogen replication. However, a more recent study using 55 MG strains collected over time since the pathogen emerged in house finches found the higher transmission rates of more recent, virulent strains could not be explained by within-host pathogen loads, suggesting instead that direct benefits to virulence in the form of tissue inflammation are more important for transmission in this system [13]. The potential importance of inflammation in mediating transmission benefits for MG in this system was further supported by recent work using a single MG strain, which found that the relative degree of conjunctival pathology (while controlling for pathogen load) among infected hosts was highly predictive of MG spread to cagemates [26].

Together, past results in the house finch-MG system suggest that the conjunctival inflammation associated with virulence may directly facilitate the likelihood of spreading a given unit of pathogen load, a phenomenon that may occur broadly for pathogens where transmission is associated with inflammation [4,6]. Further, host behaviours associated with high virulence may also augment pathogen ‘spreadability’, if hosts infected with more virulent strains spend more time in contact with bird feeders, a metric shown to influence MG transmission in experimental epidemics and free-living birds [22]. Thus, both inflammatory and behavioural mechanisms of virulence may be important drivers of higher spreadability for virulent pathogen strains, both for the house finch-MG system and more broadly. Nonetheless, empirically isolating direct effects of strain virulence on ‘spreadability’ requires methods that can directly assay relevant proxies of spread that are not confounded by potential differences in the amount of pathogen available for shedding among strains or treatments.

To address this challenge, we applied equivalent amounts of an inert, UV-fluorescent powder to ‘index birds’ experimentally inoculated with MG strains of distinct virulence or control media (table 1) to test whether virulence treatment is associated with the degree of within-flock powder spread (our metric of ‘spreadability’). To measure spreadability in a manner most relevant to MG transmission, fluorescent powder was applied directly around the conjunctivae of index birds and spreadability was quantified as the degree of powder spread to the conjunctival region of each index bird's flockmates. Any detected differences in powder spread (spreadability) across treatments thus represent both 1) how much of the starting amount of powder (equivalent for all index birds) was shed from the conjunctival region of index birds and 2) the rate of contact (both direct and indirect) between the conjunctival regions of index birds and those of their flockmates, independent of any differences in MG load. We predicted that birds experimentally infected with a high-virulence strain would spread fluorescent powder more effectively to their flockmates than those infected with a low-virulence strain or control media. We also measured potential mechanisms associated with the degree of powder spread through a flock.

**Table 1.** Experimental design for assessing associations between spreadability and virulence treatment, from no virulence (control) to infection with a high-virulence strain of *Mycoplasma gallisepticum* (MG), in house finches. Only one bird per flock (the ‘index’ bird) received the treatment, and the remaining birds (flockmates) were used to quantify powder transfer, our metric of spreadability, from the index bird at peak infectiousness (day 10 post-inoculation).

index bird treatment	no. flocks	no. birds total
control (sham inoculation)	*n* = 3	*n* = 15 (5 / group)
low-virulence MG strain	*n* = 3	*n* = 15 (5 / group)
high-virulence MG strain	*n* = 3	*n* = 15 (5 / group)

## 2. Material and methods

Forty-five hatch-year house finches were captured via cage traps in Montgomery County and the City of Radford, VA. All birds were quarantined for two weeks (see electronic supplementary material) and screened for MG seropositivity via an IDEXX MG Ab Test kit (IDEXX, Westbrook, Maine) as per [27]. After stratifying by sex to ensure equivalent sex ratios in our mixed-sex flocks (either a 2 : 3 or 3 : 2 F : M sex ratio), birds were assigned randomly to flocks (*n* = 5 birds per flock) with the exception of three seropositive birds that were assigned to control treatments given their potential prior exposure to MG (see electronic supplementary material). Each bird was given a unique combination of colour bands, one of which contained a passive integrated transponder (PIT) tag with a unique 9-digit identifier.

On experimental day −14 (i.e. two weeks prior to index bird inoculation), birds were placed in flocks in one of nine outdoor aviary units (5.5 m × 2.5 m × 2.4 m) that were set up identically (electronic supplementary material, figure S1); each flock had access to two tube-shaped feeders (one on each side of the aviary unit) containing ad libitum food (see electronic supplementary material). Each feeder had only one accessible feeding port with a perch, to which a radio frequency identification device (RFID) antenna was attached [28]. Each antenna was connected to a reader which logged any birds present each second from 06 : 00 to 20 : 00 EDT for the duration of the study. Because prior work found that the time an index bird spends on a feeder predicts the extent of transmission in experimental epidemics [22], we controlled for such variation by selecting birds from each flock that spent the second-highest amount of time on the feeder as the index bird for that group (see electronic supplementary material). The majority (7/9) of the index birds were males, but one index bird in each virulence treatment (low and high) was female.

On 9 October 2017, the selected ‘index’ bird in each flock was inoculated with 40 uL total, split between conjunctivae, of sterile Frey's medium (control treatment) or with one of two strains of *Mycoplasma gallisepticum* (*n* = 3 flocks / each) suspended in Frey's media at a concentration of 7.5 × 10^5^ colour-changing units per mL. We used the strains VA1994 (7994-1 (7P) 2/12/09), a low to mid-virulence strain and NC2006 (2006.080-5 (4P) 7/26/12), a high-virulence strain. Both strains have been repeatedly characterized in prior work and show consistent differences in virulence, as measured by the degree of conjunctival inflammation produced in house finches from the same capture population [19,24,29]. Following inoculation, we quantified clinical signs of disease twice weekly using a 0–3 scoring system described in [30], with values assigned at 0.5 intervals for clinical signs that fell intermediate to two integer scores. Scores per eye were combined at each time point to give a composite eye score ranging from 0 to 6 for each individual. Conjunctival pathogen loads were also quantified (see electronic supplementary material) over the course of infection.

**Figure 2.**
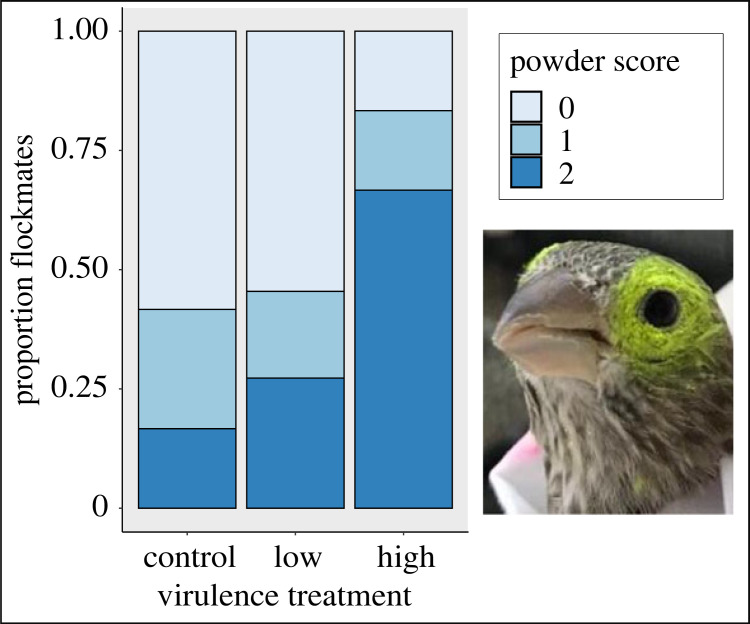
Index house finches infected with a high-virulence strain of *Mycoplasma gallisepticum* spread significantly more of the same starting amount of conjunctival UV fluorescent powder to their flockmates than did uninfected, control house finches (inset picture of an uninfected finch not used in this study, to illustrate powder application to index birds). The stacked bar chart summarizes the proportion of flockmates with a given powder score (max of score 2 observed for flockmates), representing spread from an index bird, for each treatment (*n* = 35 flockmates in 9 total groups). Scores were analysed as ordinal factors (0, 1 or 2) in a cumulative link mixed model that accounted for flockmate group as a random effect.

We used inert, UV-fluorescent powder (inset picture, figure 2) to measure the potential for a given load of MG to spread from index birds to flockmates. A single individual (R.B.) blind to treatment assignments and goals of the study (until data collection was over) used small make-up brushes to apply equivalent amounts of powder of a single colour (ECO-11 Aurora Pink; Day-Glo Colour Corp., Cleveland, OH, USA) to the feathered region directly surrounding the conjunctiva of each index bird, avoiding any direct contact with the conjunctiva itself. Powder was applied on day 10 post-inoculation (PI) to capture variation in spreadability when it is most relevant for the infectious period of MG (which peaks days 7–14; [31]). Twenty-four hours later, we captured all birds and scored the amount of powder around the conjunctiva of flockmates (within 1/2 eye width diameter of edge of the conjunctiva) as 0 = not detectable, 1 = trace amounts, 2 = moderately fluorescent or 3 = brightly fluorescent. The two conjunctivae were scored separately and summed (left plus right) within sampling day per individual for data analysis, for a maximum possible powder score of six. The same individual (R.B.) scored powder amounts for all birds while blind to treatments and study goals to prevent bias in powder application or scoring.

We used video imaging to quantify behaviours not generated by RFID data, which only record bird presence at feeding ports. We took close-up videos of a single feeder port for each flock for a minimum of 1 h per flock on days 7–9 post-inoculation (PI). Video from one flock was not usable and thus that flock was re-taped on day 16 PI. Videos were analysed by a single observer unfamiliar with the goals of the study. The number of pecks per second at food were quantified by counting the number of times an individual bird stuck its head into the port (pecks that successfully resulted in food acquisition and those that did not were considered equivalent), and dividing that total by the length of the feeding bout in seconds (defined as the total time birds were perched continuously on a feeding port).

### 2.1. Statistical analyses

All analyses were done in R v. 4.2.1 [32] using code and raw data available at [33]. To test for pairwise differences among fixed effects or their interactions in the mixed models detailed below, we used contrasts (back-transforming to the response scale) in the emmeans package [34], which calculates Tukey-adjusted *p*-values.

First, we used cumulative link mixed models (CLMM) in the ordinal package [35] to answer our core question as to whether virulence treatment predicted the degree of an index bird's powder spread to flockmates (‘spreadability’). Our predictor variable was index bird treatment (control, low-virulence, high-virulence) and we accounted for non-independence among flockmates housed with the same index bird by specifying ‘group’ as a random effect. Our response variable was the summed powder score of each flockmate (left plus right powder score), which was treated as a factor (here an ordinal factor of 0, 1 or 2; figure 2). Although powder scores had a maximum possible value of six, only the index birds (which had powder directly applied to their conjunctivae to initiate spread) were observed with summed values of six. By contrast, the maximum summed powder score for flockmates (the only birds included in the spreadability analysis), was two, reflecting the expected lower quantities of powder resulting from spread versus direct application; thus the maximum ordinal response for flockmate powder score in our CLMM was 2 (figure 2). Sex was initially included as a covariate but the parameter estimate was not significant (*p* = 0.65) and inclusion of sex did not improve the model (Likelihood ratio test: *p* = 0.65); thus sex was removed from the final model.

We next examined two potential mechanisms that may contribute to differences in spreadability among strains. To test whether virulence treatments resulted in the expected differences in severity of tissue inflammation, we tested whether conjunctivitis severity varied with index bird treatment. To eliminate non-independence in our data, we calculated the maximum eye score observed for a given index bird (*n* = 9 total) in the first two weeks PI. Because our response data did not meet the assumptions of parametric tests, we used a Kruskal-Wallis test (kruskal.test in base R) to determine whether maximum eye scores differed by treatment. We then conducted pairwise Dunn's tests using the dunn.test package [36] to determine which treatments differed significantly from each other.

Finally, we examined how virulence treatment influenced the behaviour of the experimentally infected index birds, focusing on three behavioural metrics potentially important for spreadability of MG. First, we used RFID data to quantify the length of individual feeding bouts (in seconds), defining a unique bout as >3 s long, with any RFID detection gaps at a given port greater than 4 sec long distinguishing the start of a new unique feeding bout for a given individual, as per our prior work [22]. Second, we used RFID data to quantify a proxy for the number of aggressive interactions occurring per day between focal birds and their flockmates at feeder ports, where house finches actively displace each other in competition for food resources [37]. Our proxy for aggressive interactions was the number of displacements per day at feeder ports for each bird, quantified as replacement of one individual's PIT tag by another unique PIT tag within 2 s on the same feeding port (as per [22]). As such, displacement interactions may represent opportunities for both direct or indirect contact at feeder ports. For both feeding bout length and displacement interactions, we limited our analysis to RFID data collected on days that birds were not captured and sampled (which may alter behaviour), focusing on three time-points: pre-inoculation (day −1 PI) as a baseline control, early in infection (days 1–2 PI), and at peak infectiousness for MG [31], which included the two days (days 8–9 PI) prior to powder application and the day after power quantification (day 12 PI). Finally, we used videos (collected days 7–9 PI) to collect data on the number of times per second that a bird directly pecked at food (see above).

All three analysed behavioural metrics (whether from RFID or video) contained multiple non-independent observations for a given individual; thus we used linear mixed models (LMM) or generalized linear mixed models (GLMM), implemented as lmer or glmer, respectively, in the lme4 package in R [38], with bird ID as a random effect in all models (see electronic supplementary material, for calculation of fixed effect estimate *p*-values for LMM). Foraging bout lengths (in seconds) were analysed in a GLMM with a gamma distribution and log-link function, to account for overdispersion in the integer data (see electronic supplementary material). The number of displacement interactions (total count per bird per day) were analysed in an LMM after square-root transformation, as recommended for count data [39], to meet the assumptions of linear models. Model residuals were checked for normality using Sharpiro-Wilk tests for the LMM. Data on pecks at food per second were right-skewed and did not meet the assumptions of linear models, so a gamma distribution in a GLMM was used (see electronic supplementary material). For all LMMs and GLMMs, we tested for overall significance of fixed effects using the Anova function in the car package, which generates Type II Wald chisquare tests [40].

For the RFID data models (foraging bout length, displacement interactions), we analysed the data in two ways: first, we analysed behaviour of all birds at peak infection (days 8–9 and 12 PI), testing the hypothesis that virulence treatment influenced the behaviour of index birds, but not flockmates, at peak infection. To do so, we modeled interactive effects of two categorical variables: virulence treatment (control, low, high) and bird status (index bird versus flockmate). Second, we analysed data over time for index birds only, testing the hypothesis that if MG treatment caused differences in behaviour among index birds, differences should only be present post-treatment, and strongest at peak-infection. Thus, we modelled interactive effects of virulence treatment and time period (pre-infection, early infection and peak-infection), both treated as categorical. For the number of pecks per second at food while at feeder ports, data were only analysed for index birds, and thus fixed effects included virulence treatment alone.

### 2.2. Final sample sizes

A single flockmate (from a low-virulence treatment flock) was found dead on day 10 PI from unknown causes (necropsy was unremarkable). Thus, analyses of ‘spreadability’ (data collected on day 11 PI) were limited to 35 total flockmates. A single index bird (also from the low-virulence treatment) was euthanized on day 15 PI, with necropsy results suggestive of Atoxoplasma. Thus, to ensure complete data, we limited analyses of maximum eye score for index birds to the first 14 days post-infection, when eye scores typically reach maximum values, and we limited behavioural data to the first 12 days PI. All video data were collected from complete flocks (prior to any mortality) but there were no foraging bouts recorded for a single index bird (high-virulence treatment) during video-taping. Thus, analyses of index bird behaviors from videos were limited to *n* = 8 index birds (see below). For RFID data, we had complete data from all index birds, but PIT tags of two flockmates (one from a control flock, one from a low-virulence flock) were not consistently detected by RFID antennae. Therefore, analyses of flockmate foraging bout lengths were limited to *n* = 34 flockmates.

## 3. Results

### 3.1. Spreadability to flockmates

Relative to uninfected controls, index birds that were experimentally infected with a high-virulence strain spread significantly more of the same starting amount of UV powder to flockmates (figure 2; *n* = 35; CLMM: high-virulence *β* = 2.07 ± 0.84 s.e., z = 2.46, *p* = 0.01). By contrast, experimental infection with the low-virulence strain was not associated with significantly augmented powder spread relative to controls (low-virulence *β* = 0.28 ± 0.81 s.e., z = 0.35, *p* = 0.73). *Post-hoc* contrasts found significant pairwise differences between the control and high-virulence treatments (control-high: *z* ratio = −2.46, *p* = 0.037) but only moderate and not statistically significant support for pairwise differences between the low-virulence and high-virulence treatments (low-high: *z* ratio = −2.09, *p* = 0.092). Consistent with CLMM parameter estimates, there was no support for pairwise differences between the control and low-virulence treatments **(**control-low *z* ratio = −0.35, *p* = 0.93).

### 3.2. Index bird inflammation

**Figure 3.**
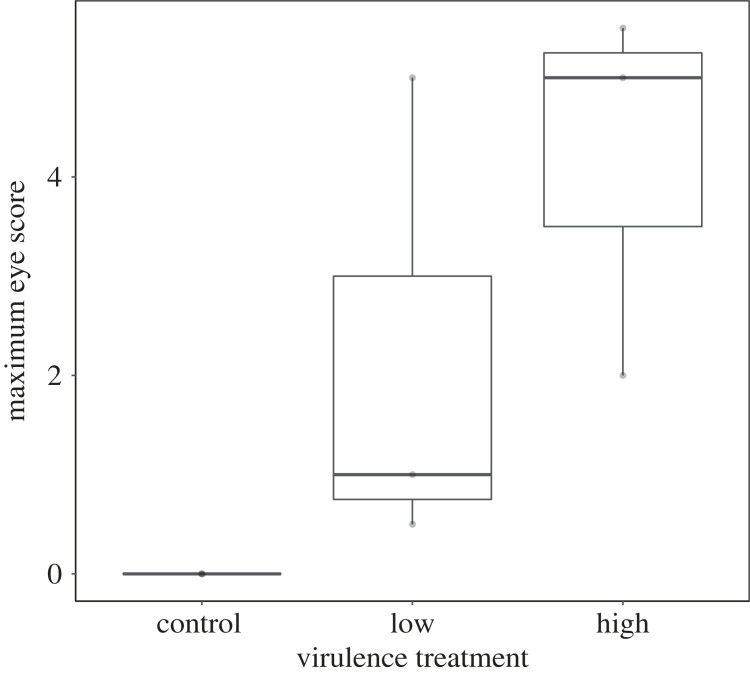
Index house finches (*n* = 9) varied significantly in the maximum eye pathology scores (left plus right scores, for a maximum value of six) observed in the first 14 days following treatment with control media, a low-virulence strain of *Mycoplasma gallisepticum*, or a high-virulence strain of *M. gallisepticum*. Darker lines represent the median, lower lines represent the 25% quartile and upper lines indicate the 75% quartile.

As expected based on our *a priori* selection of treatments known to vary in virulence, the maximum eye scores of index birds post-inoculation varied with virulence treatment (figure 3; *n* = 9, Kruskal-Wallis Chi-squared = 6.47, d.f. = 2, *p* = 0.04). Maximum eye scores were lowest, on average, for control index birds (*n* = 3; mean = 0, eye score range = 0–0), intermediate for index birds infected with the low-virulence strain (*n* = 3; mean = 2.17, eye score range = 0.5–5), and highest for index birds infected with the high-virulence strain (*n* = 3; mean = 4.17, eye score range = 2–5.5). *Post-hoc* Dunn's tests showed pairwise differences in maximum eye score between the high-virulence and control treatment (*p* = 0.018), but not the low-virulence versus control (*p* = 0.16) or low versus high-virulence treatments (*p* = 0.54).

### 3.3. Foraging bout lengths

**Figure 4.**
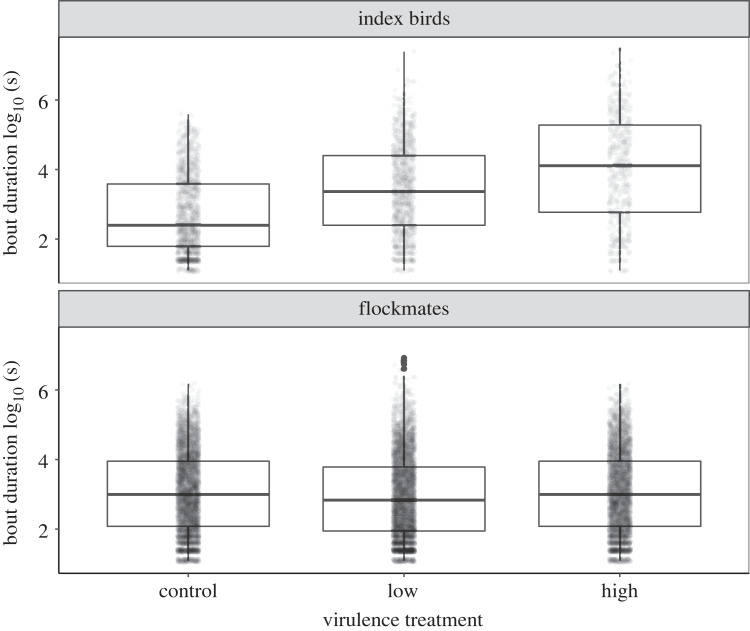
Index house finches (*n* = 9) inoculated with one of three treatments (control, low-virulence strain, or high-virulence strain of *M. gallisepticum*) varied significantly in the duration of feeding bouts (log_10_ s) at peak infection (top panel). By contrast, there were no differences in bout duration among untreated flockmates (*n* = 34 birds across 9 flocks) during the same time period (bottom panel). Each data point (jittered for visualization) represents a single unique foraging bout detected via Radio Frequency Identification Device (see *Methods*); non-independence in repeated bouts was controlled for by including bird ID as random effect. Untransformed data were analysed with a log-link function in a GLMM but are shown here log-10 transformed for ease of visualization. Box plots represent the median (dark line), 25% quartile (lower line) and 75% quartile (upper line).

Foraging bout lengths at peak infection (*n* = 16 035 unique feeding bouts from *n* = 43 unique birds over three days at peak infection [9–10 and 12 PI]) were significantly predicted by the interaction between virulence treatment and bird status, as well as a main effect of bird status, i.e. whether a bird was a directly inoculated index bird or flockmate (figure 4; status: Wald Test *χ*^2^ = 7.17; *p* = 0.007; treatment:status interaction Wald Test: *χ*^2^ = 13.1, *p* = 0.001; electronic supplementary material, table S1-S2). GLMM parameter estimates indicate that index status in interaction with both low- and high-virulence treatment was associated with significantly longer foraging bouts (figure 4, high-virulence:index status *β* = 1.54 ± 0.43 s.e., *t* = 3.58, *p* = 0.003; low-virulence:index status: *β* = 1.00 ± 0.44 s.e., t = 2.26, *p* = 0.024; electronic supplementary material, table S1). *Post-hoc* pairwise tests indicated that index birds in the high-virulence treatment significantly differed from index birds in the control treatment, as well as from flockmates in all treatment groups. However, there was no support for pairwise differences between low- and high-virulence index birds (electronic supplementary material, table S3).

When examining index birds only across time categories, there was a main effect of treatment on bout length, as well as a significant interaction between virulence treatment and time period (*n* = 4494 unique feeding bouts from nine index birds; treatment Wald Test: *χ*^2^ = 8.11, *p* = 0.017; treatment:time period Wald Test: *χ*^2^ = 180.0, *p* < 0.0001). Parameter estimates of all fixed effects show that both the low- and high-virulence strain treatments in interaction with early (for high-virulence only) and peak time period predicted longer feeding bouts for index birds (electronic supplementary material, figure S2; high-virulence:early *β* = 0.38 ± 0.19 s.e., *t* = 2.07, *p* = 0.038; high-virulence:peak *β* = 1.34 ± 0.18 s.e., *t* = 7.27, *p* < 0.0001; low-virulence:peak *β* = 1.01 ± 0.19 s.e., *t* = 5.43, *p* < 0.0001; baseline intercept [control, pre-infection]: *β* = 4.17 ± 0.28 s.e.). Pairwise contrasts found significant differences in bout lengths between high-virulence index birds and control index birds at peak infection (electronic supplementary material, table S4), as well as for high-virulence index birds at peak infection relative to earlier time points (both pre- and early infection). Interestingly, control birds also showed significant pairwise differences across time points within treatment, but while control index birds decreased in bout lengths over time, index birds in the high-virulence treatment increased in bout lengths over time (electronic supplementary material, table S4).

### 3.4. Displacement interactions

**Figure 5.**
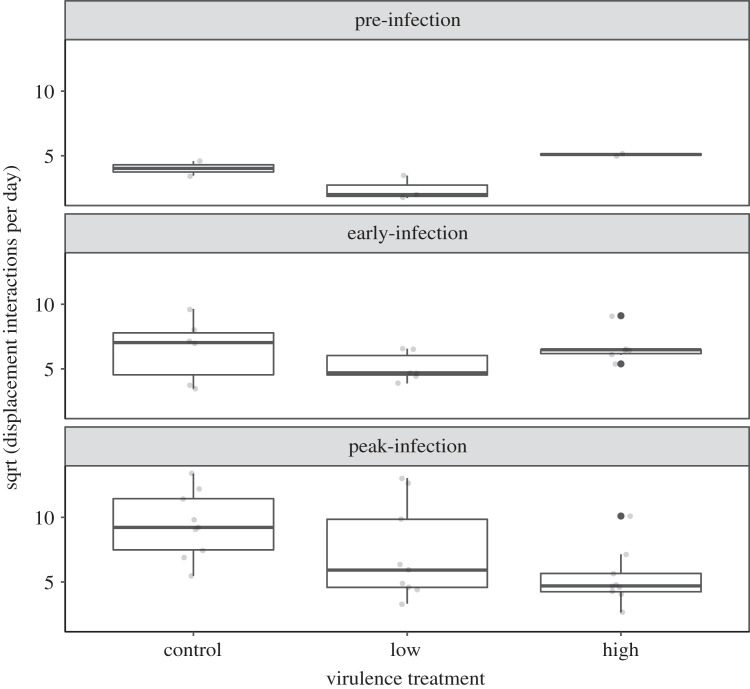
Index house finches inoculated with one of three treatments (control, low-virulence strain or high-virulence strain of *M. gallisepticum*) varied in the number of displacement interactions they had per day with flockmates over time (*n* = 52 daily values from 9 unique birds). There were no pairwise differences among treatments for any time period, but index birds in the control and low-virulence treatments showed significant changes over time (i.e. across panels), while high-virulence index birds did not. Each data point (jittered for visualization) represents a single daily sum of displacements (square root transformed to meet assumptions of linear mixed models), quantified via Radio Frequency Identification Device (see *Methods*); non-independence among individuals was controlled for in the analysis by including bird ID as random effect. Box plots represent the median (dark line), 25% quartile (lower line) and 75% quartile (upper line).

There were no detected effects of virulence treatment, bird status, or their interaction on the number of displacement interactions per day at peak infection (electronic supplementary material, figure S3; *n* = 127 daily values over 3 days from 43 unique birds; all Wald Test Effects *χ*^2^ < 2.8, *p* > 0.23). However, in an LMM of index birds only, the number of displacement interactions per day varied for index birds as a function of both time period (pre, early, peak-infection) alone, and virulence treatment in interaction with time period (figure 5; *n* = 52 daily values from *n* = 9 index birds; time period Wald Test *χ*^2^ = 37.0, *p* < 0.0001; treatment:time period interaction: Wald Test *χ*^2^ = 21.1, *p* = 0.0003; electronic supplementary material, table S5-S6). The high-virulence treatment in interaction with the peak-infection time period was associated with a significant, negative parameter estimate for the rate of displacement interactions with flockmates (high-virulence:peak *β* = −6.00 ± 1.79 s.e., *t* = −3.34, *p* = 0.002). The parameter estimate for the low-virulence treatment in interaction with peak-infection was also negative but not statistically significant (low-virulence:peak *β* = −1.88 ± 1.65 s.e., *t* = −1.14, *p* = 0.26; see electronic supplementary material, table S5 for all LMM parameter estimates including baseline intercept). Pairwise contrasts indicate that the apparent differences across treatments in the number of displacement interactions at peak infection (figure 5, bottom panel) are not statistically significant (electronic supplementary material, table S7); instead, the significant parameter estimates are driven by distinct changes in index bird displacement rates across time periods within treatment: for example, there were significant pairwise differences for index birds across time periods within both the control and low-virulence treatments, while index birds in the high-virulence treatment showed no pairwise differences over time, or relative to other treatments.

### 3.5. Pecks per second while foraging

**Figure 6.**
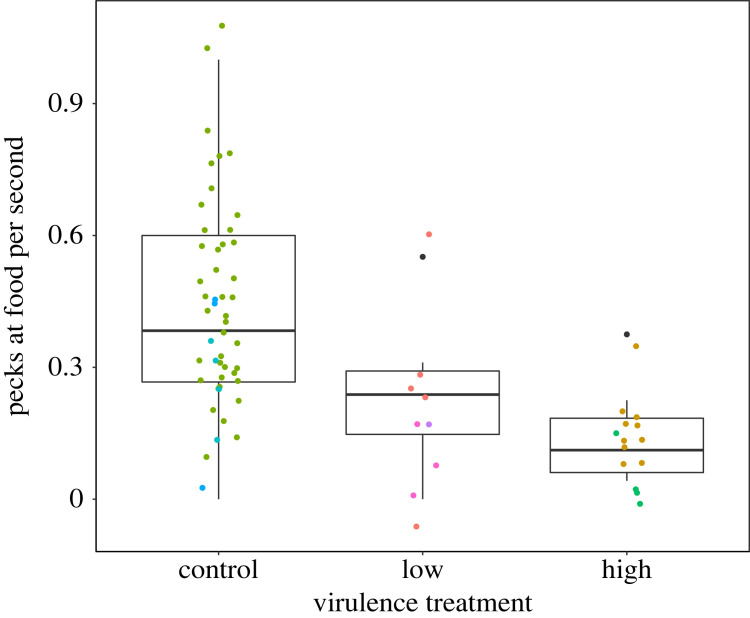
Index house finches exposed to one of three inoculation treatments (control, low-virulence strain or high-virulence strain of *M. gallisepticum*) varied significantly in the rate at which they pecked at food during peak infection, with index birds infected with the high-virulence strain of *M. gallisepticum* showing fewer pecks at food per second relative to uninfected controls during a foraging bout. Point colour represents each unique index bird (*n* = 8 for which we had video data); non-independence in repeated observations was controlled for in the analysis by including bird ID as random effect.

Virulence treatment significantly predicted the rate of pecks at food while at feeding ports (*n* = 72 observations from *n* = 8 index birds; treatment Wald Test: *χ*^2^ = 7.19, *p* = 0.028). While present at feeding ports, index birds infected with the high-virulence strain of MG made significantly fewer pecks at food per second than sham-treated control index birds (figure 6; GLMM on gamma scale with inverse parameter estimates: high-virulence *β* = 5.10 ± 2.16 s.e., *t* = 2.35, *p* = 0.018; control treatment intercept *β* = 0.38 ± 0.059). Pairwise contrasts found support only for differences between the control and high-virulence treatment (see electronic supplementary material).

## 4. Discussion

We experimentally examined whether infection virulence is associated with higher pathogen ‘spreadability’, which we define as the probability of successfully transferring a given load of pathogen (or in this case, inert fluorescent powder) to flockmates. While it can be challenging to empirically isolate potential direct benefits of virulence for pathogen transmission in non-model systems, here we paired experimental infections using treatments of distinct virulence with assays of contacts with flockmates (powder spread) that were independent of the MG load being shed by a given bird across treatments, to directly measure spreadability *per se* during peak infectiousness. By doing so, we demonstrated that birds infected with a high-virulence MG strain show higher spreadability potential than birds infected with control media, while a low-virulence strain showed no significant difference relative to the control treatment. We also explored potential mechanisms for higher spreadability in the house finch-MG system, which may relate to tissue inflammation, host behavioural changes or both.

Our primary question was whether we could measure differences in pathogen ‘spreadability’ across distinct index bird virulence treatments, from no to high virulence. Despite all index birds receiving equivalent starting amounts of inert fluorescent powder, index birds infected with a high-virulence pathogen strain were more successful at transferring the given load of powder to their flockmates than were birds inoculated with sham control media. By contrast, inoculation with the low-virulence strain did not significantly augment spreadability over control levels. Our results suggest that some characteristic associated with high-virulence, but not low-virulence, MG infections facilitates the movement of powder and thus presumably also pathogen, between hosts. However, we were not able to detect statistically significant support for pairwise differences in powder spread between our low- and high-virulence treatment groups (pairwise comparison: *p* = 0.09). One interpretation of these results is that the low-virulence treatment fell intermediate with respect to powder spread, differing significantly from neither the control nor high-virulence treatment. However, the lack of pairwise differences between the low-and high-virulence treatments also reflects sample size constraints, limiting our ability to make definitive conclusions about effects of strain virulence on spreadability. While future studies using larger sample sizes and more MG strains are needed to confirm our spreadability results, the consistency of our powder findings with the results of multiple MG transmission studies [13,24] using 3 to 55 MG strains (per study) that span a range of virulence lend support to the idea that virulent MG strains harbour direct transmission benefits. In particular, the recent study by Bonneaud *et al*. [13] leveraged variation among 55 MG strains in within-host pathogen loads to elegantly show that the benefits to high virulence in recently evolved MG strains are not pathogen-load dependent. Similarly, another recent study found that within a single MG strain, the severity of conjunctival swelling among finches is predictive of the likelihood of spread to a cagemate when pathogen load is controlled for [26]. These studies both support a role for pathogen load-independent benefits to high virulence in this system, here measured as spreadability.

Such direct benefits to high virulence for pathogens, in terms of what we refer to as spreadability, have long been hypothesized (e.g. [1]) but are difficult to isolate using empirical methods. While direct benefits of virulence for transmission can be elucidated experimentally in model systems amenable to gene knock-outs (e.g. [6,41]), or isolated statistically with sufficiently large sample sizes [13], we took a distinct approach by using powder spread to directly measure a proxy for pathogen spreadability during experimental treatments of distinct virulence. Thus, our study essentially merged experimental approaches used in other systems, whereby either powder spread is quantified during unmanipulated epidemics in free-living systems (e.g. [42]), or transmission rates are quantified across strains of distinct virulence without a paired measure of contact (e.g. [24]). By concomitantly manipulating infection virulence (none, low or high) in index hosts and measuring their ability to spread equivalent starting amounts of inert fluorescent powder, we were able to assess associations between virulence treatment and pathogen spreadability without confounding effects of variation in the amount of pathogen load available for spread among strains. At the same time, because our approach necessitated measuring spread among wild-caught birds in a free-flight setting to most closely mimic transmission dynamics for this host-pathogen system in the wild, our sample sizes and strain replication were limited. Overall, while future studies should examine a larger number of MG strains in the context of ‘spreadability’, our results combined with prior MG transmission studies [13,24,26] are strongly suggestive that strain virulence contributes to detected differences in spreadability.

A secondary goal of our study was to understand what potential mechanisms (inflammation, behavioural changes or both) contribute to the detected differences in spreadability across treatments. Importantly, effects of tissue inflammation and/or behavioural changes on spreadability could operate by facilitating shedding relevant for direct contacts (defined as close physical contacts between birds) or indirect contacts between individuals, defined here as powder deposited onto a feeder surface by an index bird and picked up by a flockmate during a later feeding bout at the same port. While our study design cannot distinguish between powder spread via direct versus indirect contacts, we can consider whether variation in the overall amount of powder spread among treatments likely resulted from differences in tissue inflammation or behavioural changes in index birds. The significant differences in conjunctival inflammation across virulence treatments (figure 3), expected based on *a priori* strain selection, mirrored our results for spreadability differences, with pairwise differences only present between control and high-virulence treatments. These results are consistent with the possibility that tissue inflammation at least partly underlies the detected differences in spreadability between the control and high-virulence treatments, though larger strain sample sizes are needed to confirm causation. Relationships between tissue inflammation and transmission have been found in other systems; for example, work by Zafar *et al*. [6,41] on the respiratory pathogen *Streptococcus pneumoniae* found that host inflammation induced by bacterial toxins during infection is key to successful host-to-host transmission. Further, prior work in house finches found that the degree of conjunctival pathology among individuals infected with the same MG strain predicted the proportion of their conjunctival pathogen load deposited onto feeder ports [43]. Thus, our among-treatment results, and those detected recently by Bonneaud *et al*. [13], are consistent with the possibility that the more severe inflammation and pathology associated with high-virulence strains in this system underlies treatment differences in spreadability, potentially due to deposition onto and resulting indirect contacts at feeders as shown in [43]. However, whether differences in conjunctival inflammation are sufficient to explain the detected differences in spreadability between the control and high-virulence treatments in our study is challenging to determine with our limited sample sizes.

Notably, we also found behavioural differences that may contribute to spreadability in this system. In particular, index birds infected with the high-virulence strain spent significantly longer bouts of time on bird feeders relative to controls. Because variation in time on feeders was associated with MG spread in experimental epidemics [22], the longer observed feeding bouts may also facilitate powder deposition onto feeding ports. On the other hand, relative to uninfected controls, birds infected with the high-virulence strain pecked at a significantly lower rate at food during a given feeding bout. Reduced feeding efficiency has also been documented in free-living house finches with conjunctivitis relative to clinically healthy birds [44], though here we only detected significantly lower peck rates at food for index birds infected with the high-virulence, but not low-virulence, strain relative to uninfected controls. While past work in this system has not directly examined whether strain virulence is associated with higher degrees of behavioural morbidity, our results and the few other systems where virulence and behavioural changes have been explicitly studied [10] are consistent with the possibility that high-virulence strains result in more extreme behavioural morbidity. If pecks at food are important opportunities for contact with feeder surfaces and resulting MG deposition, the lower food peck rates in the high-virulence treatment relative to controls should reduce rather than augment powder spread. In addition, index birds infected with the high-virulence strain were involved in significantly fewer displacement interactions with healthy flockmates than were uninfected control index birds, which may further reduce powder spread at high-virulence if displacement events result in direct or indirect contacts between hosts. However, past work manipulating feeder density in aviary units identical to those used here suggests that such displacement interactions may not contribute meaningfully to MG transmission in this system [45]. Overall, the behavioural morbidity detected in index birds in the high-virulence treatment is consistent with documented behavioural outcomes of MG infection in house finches, including lethargy, longer feeding bouts, and reduced displacements at feeders relative to healthy controls [44,46]. Importantly, at least some components of behavioural morbidity detected during infection with the high-virulence strain (reduced peck rates and displacement interactions) here would be predicted to dampen rather than augment pathogen spreadability, and thus are unlikely to underlie the detected spreadability differences between high-virulence and control treatments in our study. In support of this possibility, prior work using a single MG strain found that finches that showed stronger behavioural anorexia during infection, when controlling for associated variation in pathogen load, were less likely to spread MG to a cagemate than individuals exhibiting less severe anorexia [26].

Taken together, our results paired with past work in this system [13] suggest that inflammatory mechanisms are most likely driving the higher detected spreadability for birds infected with high-virulent MG strains relative to control birds. However, the longer amounts of time spent on feeding ports while infectious may also contribute to spreadability by providing opportunities for powder deposition onto bird feeders. Disentangling the relative contributions of inflammation, behavioural changes, and their potential interaction for spreadability would require large sample sizes, but is an important avenue for future work in this system and others. For example, in an epidemiological study of humans with influenza-less illness, Van Kerckhove *et al*. [47] found that symptomatic, but not asymptomatic, individuals altered their behaviour in ways that significantly reduced their contact rates with conspecifics, akin to house finches in this study. Nonetheless, humans with symptomatic influenza-like illness were estimated to be 3–12 times more infectious per contact than asymptomatic hosts, and were therefore predicted to contribute disproportionately to influenza transmission despite their drastically reduced contact rates [47]. Whether the high estimated infectiousness of symptomatic humans in this study was a result of higher pathogen loads in symptomatic versus asymptomatic hosts, or aspects of spreadability such as coughing or sneezing that facilitated spread of a given amount of influenza from symptomatic hosts, was not determined. However, such studies suggest that even when aspects of virulence such as tissue inflammation and behavioural morbidity have some opposing effects on spreadability as appears the case in house finches, intermediate to high levels of virulence can still be favoured if the spreadability benefits of tissue inflammation to pathogens outweigh the transmission costs associated with behavioural morbidity [10].

Although our study took place in an aviary setting, potential associations between tissue inflammation and spreadability are expected to play out similarly in the wild, where house finches commonly congregate at shared feeding spaces such as feeders [48]. Free-living house finches with conjunctivitis show similar patterns of behavioural morbidity as we found here, with symptomatic individuals spending longer time on feeders than asymptomatic birds in the wild [44]. Further, the MG strains isolated from free-living house finches since the pathogen's emergence have been increasing in average virulence over time [18,19], measured as the degree of conjunctival inflammation produced in hosts of similar genetic background. That the severity of conjunctival inflammation caused by MG has increased over time, despite higher predicted mortality rates in finches with more severe conjunctivitis [20,49], suggests that the degree of conjunctival inflammation has some adaptive benefit for MG that outweighs associated mortality costs for the pathogen in the wild. While other factors such as incomplete host immunity and the evolution of host resistance are likely contributing to virulence evolution in this system [18,29], our results and those of others [13] suggest that high virulence in MG is also favoured by the direct transmission benefits to pathogens of tissue inflammation.

Overall, understanding the extent to which virulence carries direct transmission benefits to pathogens is key to predicting the evolution of virulence both in systems where virulence carries associated benefits in terms of within-host pathogen replication [8], and in cases where host replication rate and virulence may evolve independently [50]. Direct transmission benefits of high virulence for pathogens are likely present for a breadth of host-pathogen systems where transmission success is facilitated by host tissue inflammation, regardless of whether the relevant tissue is intestinal, genital, respiratory, or conjunctival (e.g. [41,51,52]). Nonetheless, outside of the house finch-MG system, few empirical studies have explored the role of pathology-associated transmission on virulence evolution *per se*, with work to date limited to studies among diverse types of pathogens rather than among strains of the same pathogen. For example, Leggett *et al*. [53] considered 61 human pathogens that they categorized as either those where symptoms are likely to aid transmission (potentially akin to house finches and MG), symptoms are likely to inhibit transmission, or neither. By contrast to their predictions, human pathogens where symptoms are likely to augment transmission did not harbour higher average virulence relative to those systems where symptoms have no effect or even hinder transmission [53]. These results do not support a strong role for direct spreadability benefits to pathogens in driving virulence evolution, at least for the examined human pathogens. However, the challenges inherent in disentangling host and pathogen contributions to virulence can preclude our ability to uncover relationships between symptom-mediated transmission and virulence. For example, the ability of hosts to minimize virulence during infection (termed 'tolerance') will have key implications for pathogen evolution, particularly when pathology is critical for transmission success [14], yet the degree of host pathology expressed during infection is often strongly influenced by host responses [54]. Thus, understanding when and where pathogens benefit directly from causing high pathology in their hosts, and the degree to which such pathology is under pathogen versus host 'control' [54], is critical for predicting host-pathogen coevolution and the types of systems where high virulence will be favoured for pathogens.

In conclusion, our results suggest that the inflammation associated with high-virulence infections may directly facilitate pathogen spreadability, and thus provide a key fitness benefit favouring high pathogen virulence, as shown by Bonneaud *et al*. [13] for MG in house finches. Further, while not captured by our powder assay here, the exudate and pus associated with some high-virulence infections such as MG may also augment pathogen durability in the environment, leading to associations between virulence and environmental survival akin to those documented for respiratory pathogens of humans by Walther and Ewald [55]. Overall, any direct benefits associated with higher virulence in terms of strain spreadability and/or environmental durability may act in concert with higher within-host pathogen loads to facilitate the higher detected transmission rates of virulent strains in this system (e.g. [24]) and others (reviewed in [8]). Such direct benefits of virulence are often not explicitly accounted for in classic trade-off models of virulence evolution (e.g. [8,11]), but are likely to be present and important for diverse types of pathogens where successful transmission is associated with the same pathology as is virulence.

## Data Availability

All data and code are available from the Dryad Digital Repository: https://doi.org/10.5061/dryad.95x69p8ph [33]. Electronic supplementary material is available online [56].
